# L‐BAIBA Synergizes with Sub‐Optimal Mechanical Loading to Promote New Bone Formation

**DOI:** 10.1002/jbm4.10746

**Published:** 2023-04-24

**Authors:** Matt Prideaux, Alberto Smargiassi, Gang Peng, Marco Brotto, Alexander G Robling, Lynda F Bonewald

**Affiliations:** ^1^ Indiana Center for Musculoskeletal Health, Department of Anatomy, Cell Biology and Physiology, School of Medicine Indiana University Indianapolis IN USA; ^2^ Indiana Center for Musculoskeletal Health, Department of Medicine and Molecular Genetics, School of Medicine Indiana University Indianapolis IN USA; ^3^ Bone‐Muscle Research Center, College of Nursing and Health Innovation University of Texas‐Arlington Arlington TX USA

**Keywords:** ANALYSIS/QUANTITATION OF BONE, BONE HISTOMORPHOMETRY, BONE‐MUSCLE INTERACTIONS, CELLS OF BONE, OSTEOCYTES, SYSTEMS BIOLOGY‐BONE INTERACTORS

## Abstract

The L‐enantiomer of β‐aminoisobutyric acid (BAIBA) is secreted by contracted muscle in mice, and exercise increases serum levels in humans. In mice, L‐BAIBA reduces bone loss with unloading, but whether it can have a positive effect with loading is unknown. Since synergism can be more easily observed with sub‐optimal amounts of factors/stimulation, we sought to determine whether L‐BAIBA could potentiate the effects of sub‐optimal loading to enhance bone formation. L‐BAIBA was provided in drinking water to C57Bl/6 male mice subjected to either 7 N or 8.25 N of sub‐optimal unilateral tibial loading for 2 weeks. The combination of 8.25 N and L‐BAIBA significantly increased the periosteal mineral apposition rate and bone formation rate compared to loading alone or BAIBA alone. Though L‐BAIBA alone had no effect on bone formation, grip strength was increased, suggesting a positive effect on muscle function. Gene expression analysis of the osteocyte‐enriched bone showed that the combination of L‐BAIBA and 8.25 N induced the expression of loading‐responsive genes such as *Wnt1*, *Wnt10b*, and the TGFb and BMP signaling pathways. One dramatic change was the downregulation of histone genes in response to sub‐optimal loading and/or L‐BAIBA. To determine early gene expression, the osteocyte fraction was harvested within 24 hours of loading. A dramatic effect was observed with L‐BAIBA and 8.25 N loading as genes were enriched for pathways regulating the extracellular matrix (*Chad*, *Acan*, *Col9a2*), ion channel activity (*Scn4b*, *Scn7a*, *Cacna1i*), and lipid metabolism (*Plin1*, *Plin4*, *Cidec*). Few changes in gene expression were observed with sub‐optimal loading or L‐BAIBA alone after 24 hours. These results suggest that these signaling pathways are responsible for the synergistic effects between L‐BAIBA and sub‐optimal loading. Showing that a small muscle factor can enhance the effects of sub‐optimal loading of bone may be of relevance for individuals unable to benefit from optimal exercise. © 2023 The Authors. *JBMR Plus* published by Wiley Periodicals LLC on behalf of American Society for Bone and Mineral Research.

## Graphical abstract

The muscle metabolite L‐BAIBA synergizes with sub‐optimal mechanical loading of the mouse tibia to promote new bone formation and regulate the transcriptional response of osteocyte‐enriched cortical bone.

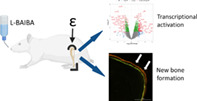

## Introduction

A fully functioning musculoskeletal system depends upon the coordinated activities of muscle and bone. While physical interactions between muscle and bone are required for the movement of the skeleton, muscle and bone cells are also known to interact at the molecular level through the secretion of hormones and small molecules.^(^ ^1^, ^2^ ^)^ Osteoblasts and osteocytes secrete molecules such as prostaglandin E2 (PGE_2_)^(^ ^3^ ^)^ and proteins such as Wnt1, Wnt3a,^(^ ^4^ ^)^ and osteocalcin,^(^ ^5^ ^)^ which promote myogenesis in vitro and in vivo. On the other hand, muscle cells can secrete factors that act on bone cells to elicit either positive or negative effects on bone homeostasis, depending on whether the muscle is contracted or flaccid. Examples of these muscle‐secreted molecules (termed myokines) include interleukin 6 (Il‐6),^(^ ^6^, ^7^ ^)^ myostatin, a negative regulator of muscle and bone,^(^ ^8^, ^9^ ^)^ and irisin^(^ ^10^, ^11^ ^)^ and β‐aminoisobutyric acid (BAIBA),^(^ ^12^, ^13^ ^)^ which are thought to have positive effects on bone. In particular BAIBA, a metabolite derived from the catabolism of valine or thymine, is elevated in plasma in response to exercise.^(^ ^12^, ^14^, ^15^ ^)^ BAIBA release is under the control of peroxisome proliferator‐activated receptor gamma coactivator 1‐alpha (PGC‐1α), a key regulator of the adaptive response to endurance exercise, mitochondrial biogenesis, and energy metabolism.^(^ ^16^, ^17^ ^)^ BAIBA naturally occurs as two enantiomers, L‐BAIBA and D‐BAIBA (also known as R‐BAIBA and S‐BAIBA, respectively). While many of the previous studies investigating the functions of BAIBA did not differentiate between these two isoforms, more recent studies have identified differences in the abundance and functions of the enantiomers in different tissues and under physiological and pathological conditions.^(^ ^13^ ^)^

BAIBA targets several tissues, including the liver^(^ ^18^ ^)^ and adipose tissue.^(^ ^12^ ^)^ We reported previously that L‐BAIBA had protective effects on maintaining muscle and on bone mass through its actions on osteocytes.^(^ ^13^ ^)^ As the most numerous and long‐lived bone cells, osteocytes play essential roles in regulating bone homeostasis. L‐BAIBA protects osteocytes from the damaging effects of reactive oxygen species (ROS) on mitochondrial dysfunction and, ultimately, osteocyte cell death/apoptosis. Interestingly, L‐BAIBA was over 100 times more potent than D‐BAIBA in exerting these anti‐apoptotic effects, which points to a specific role for L‐BAIBA in protecting osteocytes and further shows the importance of analyzing the effects of these enantiomers separately.^(^ ^13^ ^)^

One of the most important and well‐described functions of osteocytes within bone is as a mechanosensory cell type.^(^ ^19^, ^20^ ^)^ Osteocytes sense mechanical load placed on bone during exercise and respond by regulating the synthesis and secretion of biochemical signals that target osteoblasts and osteoclasts to regulate bone formation and resorption, respectively. In particular, osteocytes are crucial for the immediate response to loading, where they rapidly release small signaling molecules such as Ca^2+^, PGE_2_, nitric oxide (NO), and ATP.^(^ ^21^, ^22^, ^23^ ^)^ This initial response is followed by the downregulation of proteins such as the secreted Wnt/β‐catenin pathway inhibitors sclerostin and DKK1,^(^ ^24^ ^)^ leading to increased osteoblast activity and bone formation. The importance of mechanical loading on maintaining bone health has been demonstrated by hind limb suspension studies, a well‐described model of disuse where the inability to load the bone results in decreased bone mass and strength.^(^ ^13^ ^)^ We previously found that administration of L‐BAIBA in drinking water not only protected male and female C57Bl/6 mice against hind limb suspension‐induced bone loss but also against muscle loss, suggesting the paracrine activity of L‐BAIBA on muscle.

The muscle‐bone crosstalk through the release of myokines is known to regulate the function of bone cells such as osteoblasts and osteocytes.^(^ ^1^ ^)^ As L‐BAIBA is known to be increased by exercise, we hypothesized that a potential function of L‐BAIBA was to act on the mechanically loaded bone during exercise to potentiate the anabolic effects of loading. Therefore, in this study we investigated the anabolic response of bone in mice treated with L‐BAIBA and subjected to a tibial mechanical loading regime that was insufficient to promote bone formation alone (sub‐optimal loading). We found that bone formation in mice subjected to sub‐optimal loading was only significantly induced in mice also administered L‐BAIBA. Furthermore, via RNA‐sequencing (RNA‐seq) analysis we identified several enriched pathways and genes in osteocyte‐enriched bone that showed early (24 hours after loading) and late (after 2 weeks) responses to stimulation by L‐BAIBA and/or sub‐optimal mechanical loading. Our results have important implications for how muscle and bone function cooperatively during exercise and the mechanisms by which myokines promote bone health.

## Material and Methods

### Animals

Mice were obtained from the National Institute on Aging (NIA, Bethesda, MD, USA) and housed at the Indiana University Animal Care Facility. C57BL/6 male mice 5 months of age were selected because at this age mice are skeletally mature and have passed the rapid phase of bone growth that occurs during development.^(^ ^25^ ^)^ Mice were housed four to five per cage under standard conditions for 1–2 weeks before they were single‐caged and used in the experiment. Three days prior to the beginning of the experiment the mice were separated into groups; L‐BAIBA (100 mg/kg/day, AdipoGen Life Sciences) was provided in drinking water ad libitum to the treated group, while control groups were provided with regular water. The mice were fed regular chow (2018 Teklad Global 18% Protein Extruded Rodent Diet, ENVIGO) and water ad libitum and maintained at a constant temperature of 25°C on a 12‐hour light/dark schedule. Water intake, weight, and overall health of the animals were monitored and documented during the total duration of the experiment. Protocols were approved by the Institutional Animal Care and Use Committee (IACUC) of Indiana University School of Medicine.

### In vivo loading

The effects of short‐ and long‐term L‐BAIBA administration on the anabolic response to sub‐optimal mechanical loading were assessed using the in vivo tibial axial loading model. Mice were anesthetized (2%–3% isofluorane) and their right tibiae subjected to axial compression under continuous anesthesia for 220 cycles using a sinusoidal waveform of 2 Hz with no intercycle rest period (EnduraTEC ELF 3200; Bose, Massachusetts, USA). We selected age‐specific peak forces in order to generate peak periosteal strains between −1500 and − 2200 με at the cortical midshaft, which resulted in magnitudes of 7 and 8.25 N applied to the tibia (*n* = 6–8 per group). These values were determined from previously performed strain gaging of C57Bl/6 male mice.^(^ ^26^ ^)^ After each loading session the mice were returned to their cages, where they were observed to resume unrestricted activity. The left tibia served as a contralateral nonloaded control.

**Fig. 1:**
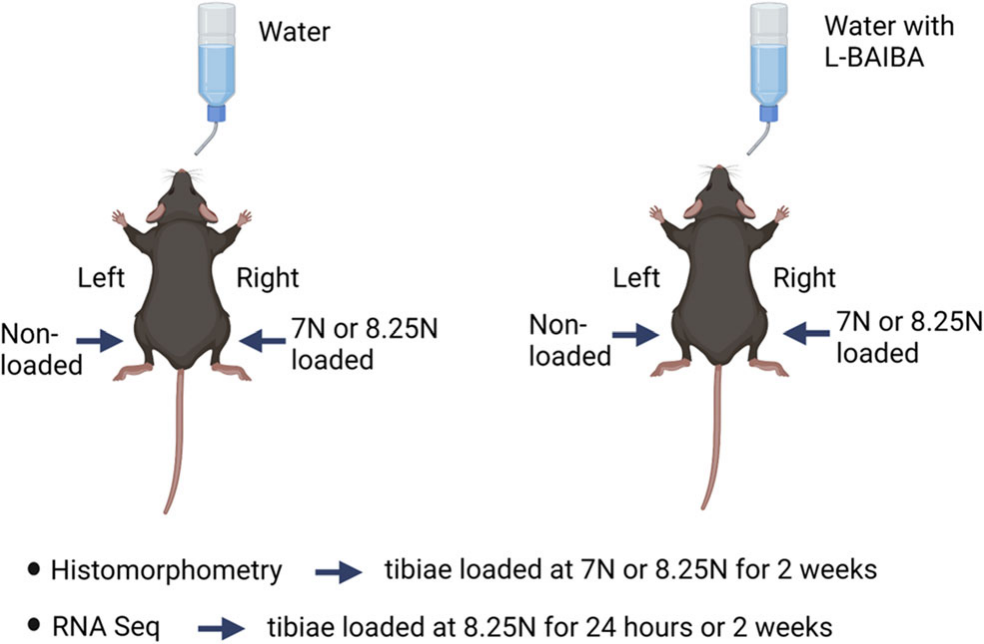
Experimental design of tibial loading and L‐BAIBA administration. For the analysis of bone formation by dynamic histomorphometry, mice were given untreated water (vehicle) or water supplemented with 100 mg/kg/day L‐BAIBA for 2 weeks. The right tibia was loaded three times per week with 7 N or 8.25 N force and the left tibiae served as nonloaded controls (*n* = 6–8 per group). For RNA‐seq analysis of the bone transcriptome, mice were given untreated water or 100 mg/kg/day L‐BAIBA for 5 days, and the right tibia was harvested 24 hours after a single bout of loading at 8.25 N. To examine the effects of a longer L‐BAIBA and loading treatment duration, mice were given untreated water or 100 mg/kg/day L‐BAIBA for 2 weeks, and the right tibia was subjected to three bouts of 8.25 N loading per week. To ensure sufficient RNA yield, the tibiae from two mice were combined to provide one sample (*n* = 2–3 samples per group). Created with BioRender.com.

To examine the bone formation response to L‐BAIBA and sub‐optimal loading, 5‐month‐old male C57Bl/6 mice were loaded three times per week for 2 weeks at either 7 or 8.25 N (Fig. 1). L‐BAIBA (100 mg/kg/day) was provided in the drinking water throughout the 2‐week loading period. To examine transcriptional changes in the bone in response to L‐BAIBA and sub‐optimal loading, 5‐month‐old male mice were loaded at 8.25 N three times per week for 2 weeks as described earlier (long term) or pretreated with L‐BAIBA for 5 days prior to a single bout of 8.25 N loading (short term) (Fig. 1). To ensure enough RNA for sequencing, the tibiae from two mice were combined to give one sample (*n* = 2–3 samples per group). For both experiments, the mice were sacrificed 24 hours after the final application of loading and the tibiae dissected.

### Dynamic histomorphometry

Two fluorochromes were used to label and track bone formation using dynamic histomorphometry of the bones; Calcein green (10 mg/kg; Sigma, Saint Louis, MO, USA) was injected intraperitoneally the first day of loading, and Alizarin complexone (30 mg/kg; Sigma) was injected the last day of tibial loading. Mice were sacrificed 4 days after the last tibial compression. Both loaded and unloaded tibiae were collected and fixed in 4% paraformaldehyde. After 48 hours, the fixative was removed and substituted with 70% EtOH. The tibiae were then dehydrated through graded EtOH (70–100%), cleared in xylene, infiltrated with unpolymerized methyl methacrylate (MMA), and embedded in polymerized MMA for subsequent sectioning. To measure bone formation, dynamic histomorphometric analysis was performed using a Lionheart FX Automated Microscope (BioTek Instruments, Winooski, VT, USA) and ImageJ (National Institutes of Health [NIH], https://imagej.nih.gov/ij/) on 20‐ to 40‐μm‐thick sections taken approximately 3 mm proximally to the bony connection at the tibiofibular junction, as described in Lewis et al.^(^ ^26^ ^)^ Measured parameters included mineralizing surface (MS/BS), mineral apposition rate (MAR), and bone formation rate (BFR/BS). For each section, we analyzed the entire endocortical (Ec) and periosteal (Ps) surfaces separately. Analyses were performed on the entire cross section of bone. To determine the relative bone formation parameters, the values from the left, nonloaded tibia were subtracted from the right, loaded tibia, as previously described.^(^ ^26^ ^)^

### Grip strength

The day before the sacrifice, absolute grip strength was tested in all animals using a grip strength meter (Columbus Instruments, Columbus, OH, USA). Five measurements were registered per animal, and the mean of the top three measurements was calculated. Results were then normalized to body weight to give the relative grip strength (g/gr).

### Serum aminobutyric acid measurements

Blood was collected by cardiac puncture the day of the sacrifice. Approximately 1 mL of blood per animal was collected in blood collection tubes (Becton, Dickinson and Company, Franklin Lakes, NJ, USA). Blood was then centrifuged at 1000 RPM for 10 minutes to separate the serum. The obtained serum was then analyzed to identify the presence of aminobutyric acids using liquid chromatography‐mass spectrometry (LC–MS) as described in Wang et al.^(^ ^27^ ^)^ Briefly, standard calibration curves were prepared by spiking the pure standards in surrogate matrix (5% bovine serum albumin‐PBS). 10 μL of serum was mixed with 10 μL internal standards mixture solution (1.2 μM *D*,*L*‐BAIBA‐d_3_ in 0.1% formic acid in methanol, v/v) and 35 μL of 0.1% formic acid in methanol (v/v). Then the mixtures were shaken for 20 minutes at room temperature, followed by centrifugation at 12,000 × *g*, 4°C, for 15 minutes to precipitate protein. Finally, 45 μL of supernatant from each sample was injected for LC–MS/MS analysis.

LC was performed using pumps A and B (LC‐30 AD) and autosampler (SIL‐30 AC) (Shimadzu Scientific Instruments, Tokyo, Japan). The LC separation was conducted on a chiral SPP‐TeicoShell, 150 × 4.6 mm, 2.7‐μm column (AZYP LLC, Arlington, TX, USA) configured with a Max‐RP 50 × 2.0 mm as guard column (Phenomenex, Torrance, CA, USA). The analytical column was maintained at room temperature. The mobile phase consists of methanol (A) and buffer (B) containing 0.005% formic acid and 2.5 mM ammonium formate in water. The LC gradient program was as follows: 0.0–10 minutes, 25% B; 10–10.1 minutes, 25–98% B; 10.1–17.0 minutes, 98% B; 17.1–25 minutes, 25% B. The flow rate was 0.6 mL/min.

The LC–MS/MS analysis was performed on a Shimadzu LCMS‐8050 triple quadrupole mass spectrometer. The instrument was operated and optimized under positive electrospray and multiple reaction monitoring modes (+ESI MRM) using pure standard solutions. The optimized conditions were as follows: interface voltage, 4.0 kV; interface temperature, 300°C; desolvation line temperature, 300°C; heating block temperature, 400°C; drying gas (N_2_), 10 L/min; nebulizing gas (N_2_), 3 L/min; heating gas (air), 10 L/min; collision‐induced dissociation gas (Ar), 230 kPa. All analyses and data processing were completed on Shimadzu LabSolutions version 5.91 software.

### RNA sequencing

RNA‐seq was performed to determine transcriptional changes in osteocyte‐enriched cortical bone in response to short‐ and longer‐term mechanical loading and BAIBA administration. Soft tissue and epiphyses were removed from the dissected tibiae and the marrow flushed with PBS using a 27‐gauge needle. The bones were bisected longitudinally and serially digested in collagenase and ethylenediaminetetraacetic acid (EDTA, Sigma) to remove cells from the bone surface and leave an osteocyte‐enriched cell population as described in Prideaux et al.^(^ ^28^ ^)^ Briefly, the bone pieces were digested in 2 mg/mL collagenase for 25 minutes, followed by incubation in 5 mM EDTA for 25 minutes and a final digestion in 2 mg/mL collagenase for 25 minutes. All incubations were performed in a sterile incubator at 5% CO_2_, 37°C, on an orbital shaker at 180 rpm. The bone pieces were then snap‐frozen in liquid nitrogen.

### Library preparation and sequencing

The digested cortical bone chips were pulverized in liquid nitrogen and homogenized in 1 mL Trizol (Thermo Fisher Scientific, Waltham, MA, USA). RNA was isolated using the DirectZol MicroPrep kit according to the manufacturer's instructions. The concentration and quality of total RNA samples were assessed using Agilent 2100 Bioanalyzer. All samples had a RIN (RNA Integrity Number) of four or higher. 100 ng RNA per sample were used to prepare a dual‐indexed strand‐specific cDNA library using KAPA mRNA Hyperprep Kit (Roche). The resulting libraries were assessed for quantity and size distribution using Qubit and Agilent 2100 Bioanalyzer. 200‐pM pooled libraries were utilized per flow cell for clustering amplification on cBot using HiSeq 3000/4000 PE Cluster Kit and sequenced with 2 × 75 bp paired‐end configuration on HiSeq4000 (Illumina) using HiSeq 3000/4000 PE SBS Kit. A Phred quality score (Q score) was used to measure the quality of sequencing. Over 90% of the sequencing reads reached Q30 (99.9% base call accuracy).

### Sequence alignment and gene counts

The sequencing data were first assessed using FastQC (Babraham Bioinformatics, Cambridge, UK) for quality control. Then all sequenced libraries were mapped to the mm10 mouse genome using STAR RNA‐seq aligner^(^ ^29^ ^)^ with the following parameter: “—outSAMmapqUnique 60.” The reads distribution across the genome was assessed using bamutils (from ngsutils).^(^ ^30^ ^)^ Uniquely mapped sequencing reads were assigned to mm10 refSeq genes using featureCounts (from subread)^(^ ^31^ ^)^ with the following parameters: “−s 2 −p –Q 10.” Quality control of sequencing and mapping results was summarized using MultiQC.^(^ ^32^ ^)^ Genes with read counts per million (CPM) > 0.5 in more than three of the samples were kept. The data were normalized using the trimmed mean of M (TMM) values method. Differential expression analysis was performed using edgeR.^(^ ^33^, ^34^ ^)^ False discovery rate (FDR) was computed from *p* values using the Benjamini‐Hochberg procedure. The raw and analyzed RNA‐seq data can be found at GEO (Gene Expression Omnibus) no. GSE214582.

### Statistical analysis

Data analysis was performed using GraphPad Prism 9 for Windows (GraphPad Software, La Jolla, CA, USA). Two‐way ANOVA with Tukey's post hoc test was used to compare differences between multiple variables. One‐way ANOVA with Tukey's post hoc test or Kruskal‐Wallis test with Dunn's post hoc test was used for single variable comparisons. A *p* value <0.05 was considered significant.

Gene Set Enrichment Analysis was applied on gene sets from Gene Ontology (GO)^(^ ^35^ ^)^ using the R package clusterProfiler.^(^ ^36^ ^)^ Significant genes were defined as genes with *p* value <0.05 and absolute log2 fold‐change >2. Supplemental files containing the differentially expressed genes and GO pathway analysis were deposited at Mendeley Data and are available at https://doi.org/10.17632/7ncthkxw26.1.

## Results

### L‐BAIBA administration increases serum L‐BAIBA levels and improves grip strength

**Fig. 2:**
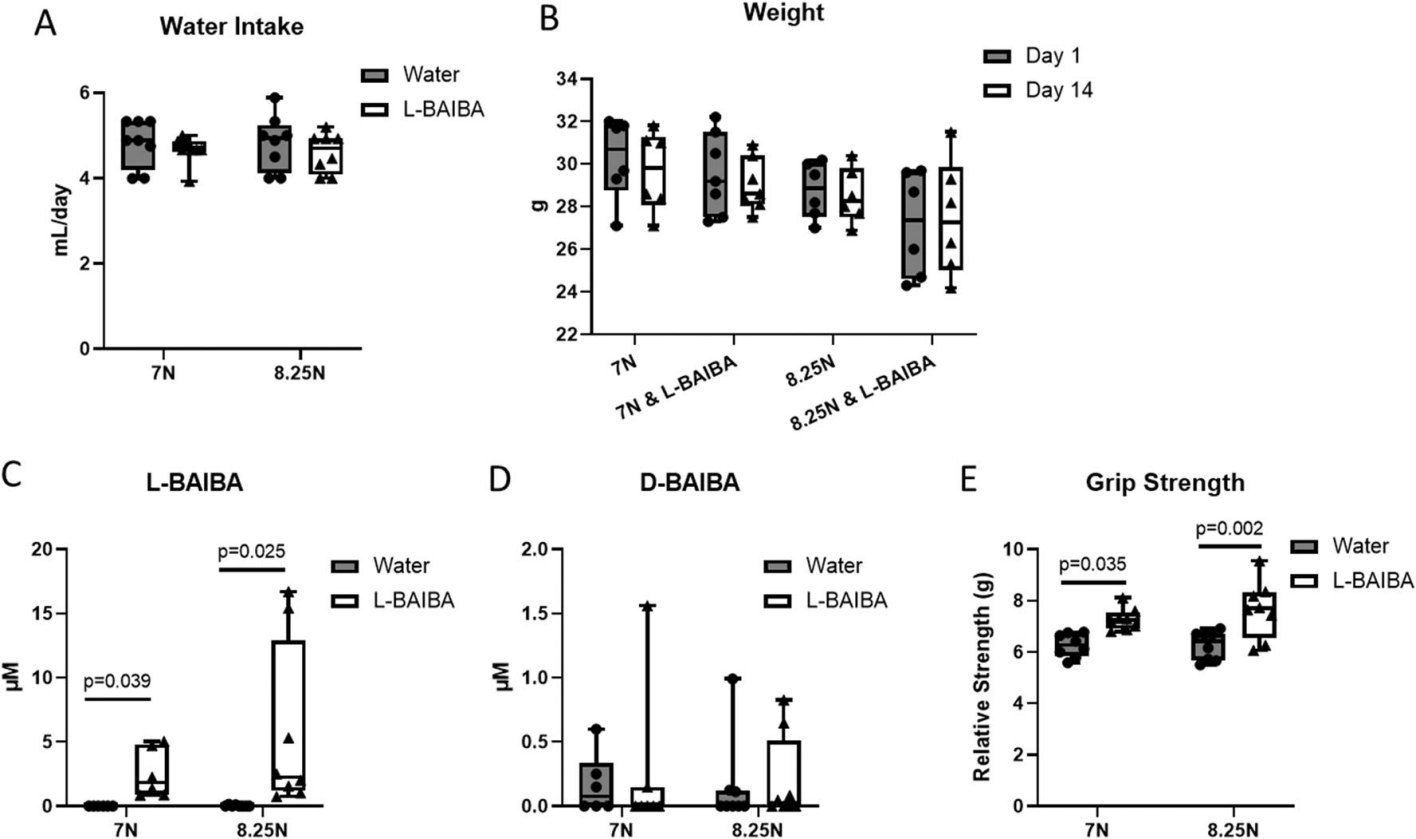
L‐BAIBA administration leads to elevated serum L‐BAIBA levels and increased muscle strength. Water intake (*A*) and body weight (*B*) were unaffected by L‐BAIBA supplementation or tibial loading. Serum L‐BAIBA levels were elevated in the mice given L‐BAIBA (*C*), but the levels of D‐BAIBA (*D*) were not significantly affected. (*E*) Grip strength was increased in the L‐BAIBA‐treated mice. Data are presented as mean ± SD, *n* = 7–8.

Since mice can be sensitive to changes in water taste due to the addition of compounds, we first examined the water intake of mice given the L‐BAIBA supplemented drinking water. Supplementation of drinking water with 100 mg/kg L‐BAIBA had no effect on water intake (Fig. 2*A*). This is consistent with our previous studies.^(^ ^13^ ^)^ We also observed no effect of L‐BAIBA administration on the weight of the mice after 14 days of L‐BAIBA treatment (Fig. 2*B*). To confirm that administration of L‐BAIBA in the drinking water of mice resulted in increased levels of L‐BAIBA in serum, we measured serum L‐BAIBA levels at the time of sacrifice by LC–MS. L‐BAIBA levels were significantly elevated in the L‐BAIBA‐treated mice compared to the mice given only water (Fig. 2*C*). Treatment with L‐BAIBA did not significantly alter the serum levels of its enantiomer D‐BAIBA (Fig. 2*D*).

To determine whether L‐BAIBA regulated skeletal muscle function, grip strength testing was performed on L‐BAIBA and control‐treated mice. L‐BAIBA increased grip strength in the mice; however, there was no difference between the mice receiving 7 or 8.25 N of load (Fig. 2*E*). These measurements suggest that L‐BAIBA is a positive regulator of muscle strength.

### L‐BAIBA or sub‐optimal loading at 7 or 8.25 N does not significantly increase bone formation

**Fig. 3:**
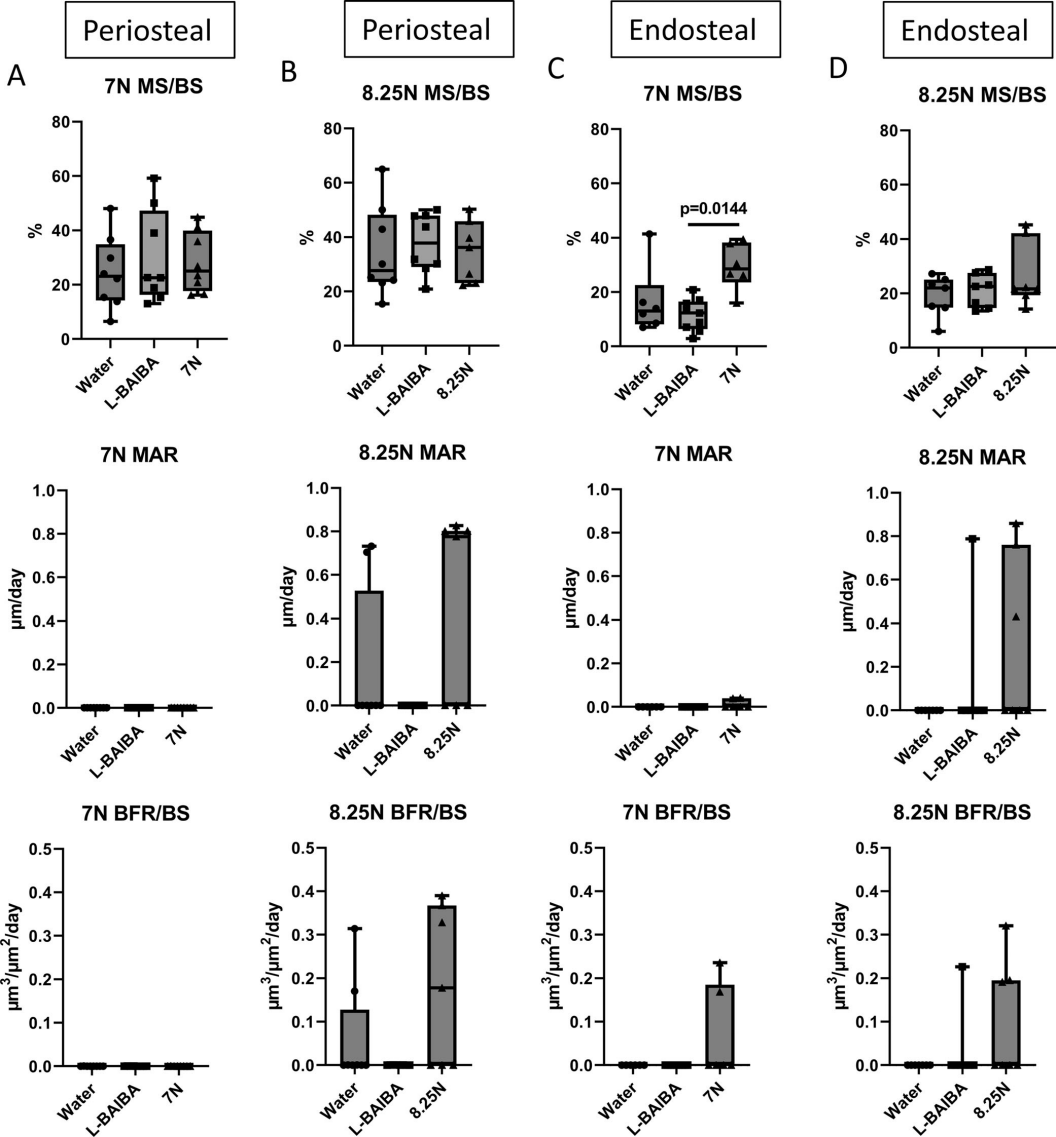
L‐BAIBA supplementation or mechanical loading alone does not induce new bone formation. Mineralizing surface/bone surface (%MS/BS), mineral apposition rate (MAR), and bone formation rate (BFR/BS) between loaded and nonloaded tibiae from vehicle‐treated (water) mice and the nonloaded tibiae from mice receiving L‐BAIBA were compared to determine whether loading or L‐BAIBA alone promote bone formation. No significant effects were observed on periosteal %MS/BS, MAR, or BFR/BS in response to L‐BAIBA, 7 N (*A*) or 8.25 N (*B*) mechanical loading. (*C*) Endosteal %MS/BS was increased by 7 N loading compared to L‐BAIBA, but there were no differences in any other parameters. (*D*) There were no significant effects of L‐BAIBA or 8.25 N loading on %MS/BS, MAR, or BFR/BS. Data are presented as mean ± SD, *n* = 6–8.

To determine the level of new bone formation induced by L‐BAIBA or sub‐optimal mechanical loading alone, we compared the ratio of mineralizing surface/bone surface (%MS/BS), mineral apposition rate (MAR), and bone formation rate (BFR/BS) between loaded and nonloaded tibiae from vehicle (water only)‐treated mice, and the nonloaded tibiae from mice receiving L‐BAIBA. L‐BAIBA administration alone did not affect periosteal or endosteal bone formation (Fig. 3*A*–*D*). No changes in periosteal MS/BS, MAR, or BFR/BS were observed in response to 7 N (Fig. 3*A*) or 8.25 N (Fig. 3*B*) mechanical loading, although there was a high degree of variability in the 8.25 N loaded group. Endosteal MS/BS was significantly increased by 7 N loading, with no effect on MAR or BFR/BS (Fig. 3*C*), and 8.25 N loading did not significantly affect endosteal MS/BS, MAR, or BFR/BS (Fig. 3*D*).

### L‐BAIBA synergizes with sub‐optimal mechanical loading to promote bone formation

**Fig. 4:**
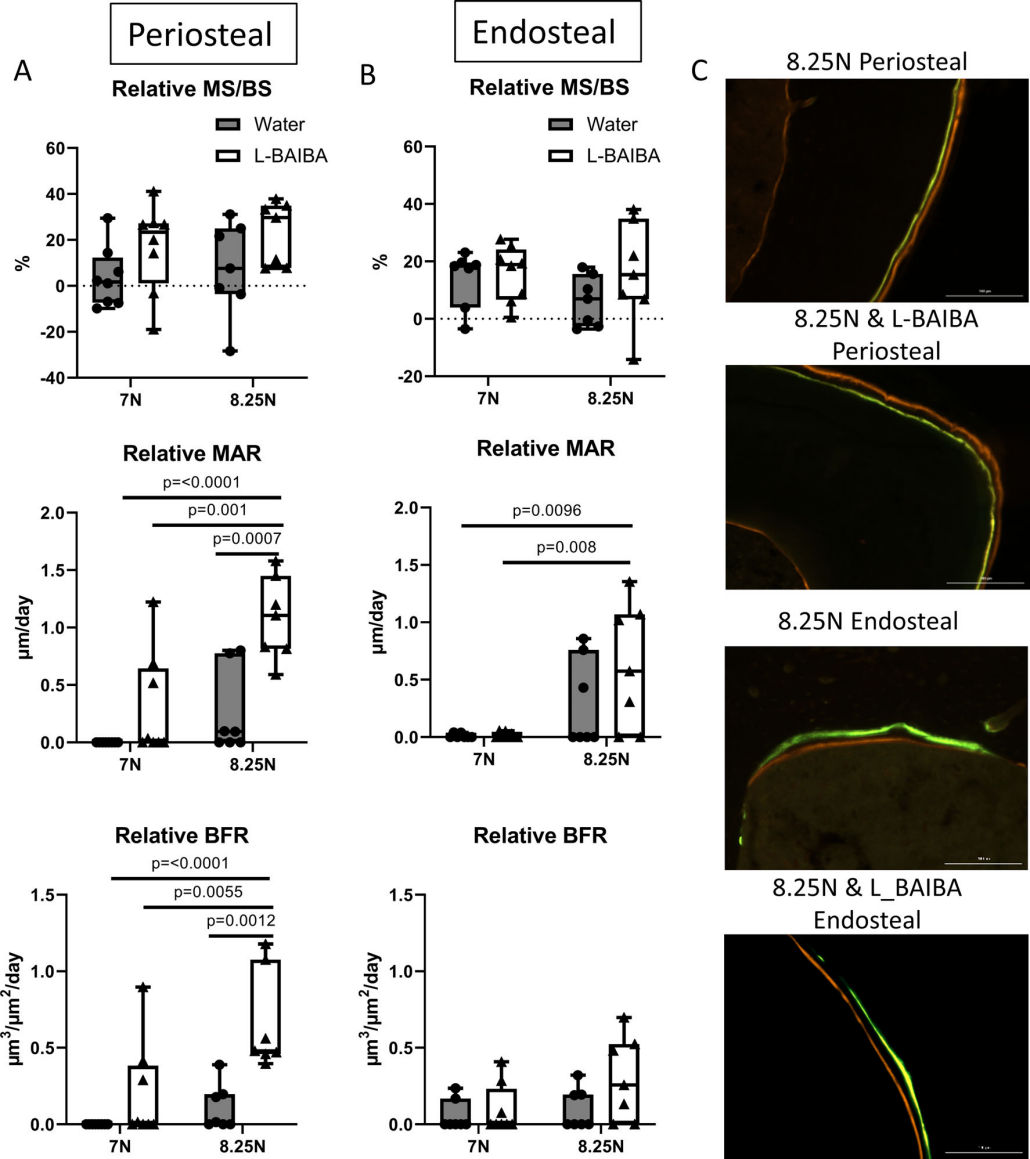
L‐BAIBA synergizes with sub‐optimal mechanical loading to promote periosteal bone formation. (*A*) Relative MAR and BFR/BS were significantly increased by the combination of L‐BAIBA treatment and 8.25 N loading compared to 8.25 N loading alone. (*B*) Endosteal bone formation parameters were not significantly enhanced by L‐BAIBA in addition to loading. (*C*) Representative images of periosteal and endosteal double‐labeled bone surfaces used to measure bone formation. Data are presented as mean ± SD, *n* = 6–8.

To determine whether L‐BAIBA administration could synergize with sub‐optimal mechanical loading to further enhance bone formation, we examined the osteogenic response in loaded tibiae from mice treated with or without L‐BAIBA. Tibial loading at a peak load of 7 N failed to increase periosteal relative MS/BS, MAR, or BFR/BS, regardless of L‐BAIBA supplementation. Likewise, tibia loading at 8.25 N was ineffective at increasing bone formation parameters in vehicle‐treated mice. However, mice loaded at 8.25 N in the presence of L‐BAIBA exhibited significantly increased periosteal MAR and BFR/BS compared to vehicle‐treated mice loaded at 7 or 8.25 N or L‐BAIBA‐treated mice loaded at 7 N (Fig. 4*A,C*). There were no significant effects of L‐BAIBA and either 7 or 8.25 N loading on endosteal bone formation parameters (Fig. 4*B,C*).

**Fig. 5:**
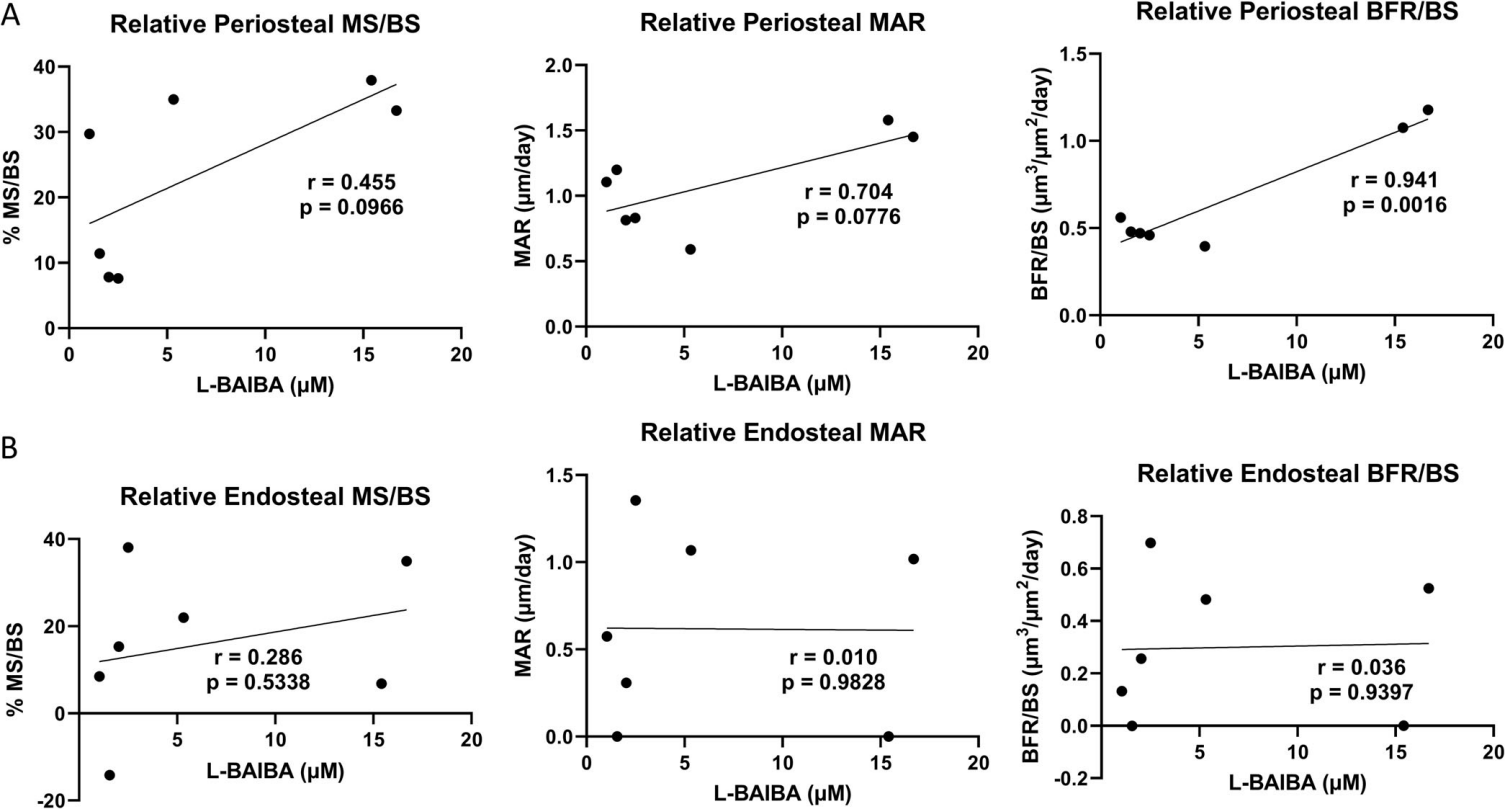
Serum L‐BAIBA levels are positively correlated with periosteal bone formation in the 8.25 N loaded mice. (*A*) There is a trend toward correlations between periosteal MS/BS and MAR and serum L‐BAIBA and a significant correlation with BFR/BS and L‐BAIBA. (*B*) There is no correlation with endosteal bone formation parameters and L‐BAIBA. *n* = 7.

To further investigate the link between L‐BAIBA and bone formation in the 8.25 N loaded mice, Pearson's correlation was performed for periosteal and endosteal bone parameters. A significant correlation of 0.941 was identified between serum L‐BAIBA levels and periosteal BFR/BS, and there was a trend toward correlations between L‐BAIBA and periosteal MS/BS and MAR (Fig. 5*A*). However, this might have been driven by the two mice with the highest serum L‐BAIBA levels, which showed a dramatically greater BFR and MAR. There were no correlations between serum L‐BAIBA and any of the endosteal bone formation parameters (Fig. 5*B*). There were also no significant correlations with serum L‐BAIBA and bone formation parameters in the 7 N loaded mice (data not shown).

### Regulation of bone transcriptome by 2 weeks of sub‐optimal loading or L‐BAIBA treatment

**Fig. 6:**
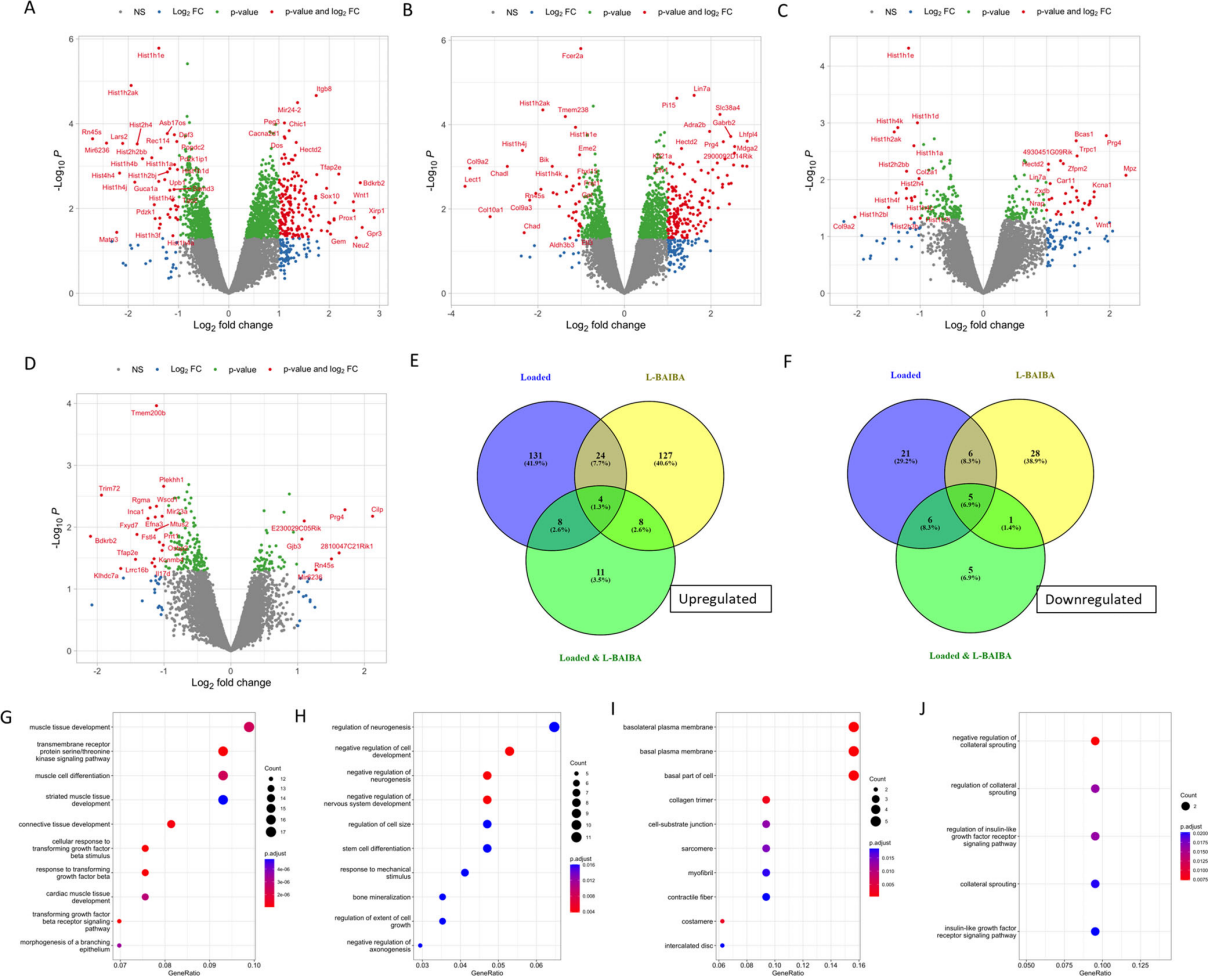
The effects of 2 Weeks of 8.25 N sub‐optimal loading and L‐BAIBA on bone transcriptome. Volcano plots showing significantly regulated genes between (*A*) vehicle‐treated loaded tibiae versus vehicle‐treated nonloaded tibiae, (*B*) L‐BAIBA‐treated nonloaded tibiae versus vehicle‐treated nonloaded tibiae, (*C*) L‐BAIBA‐treated loaded tibiae versus vehicle‐treated nonloaded tibiae, and (D) L‐BAIBA‐treated loaded tibiae versus vehicle‐treated loaded tibiae (*E*). Venn diagram showing number of significantly upregulated and downregulated (*F*) genes between loaded, L‐BAIBA and loaded, and L‐BAIBA nonloaded groups compared to vehicle‐treated nonloaded tibiae. Gene Ontology (GO) analysis of the significantly regulated genes between (*G*) vehicle‐treated loaded tibiae versus vehicle‐treated nonloaded tibiae, (*H*) L‐BAIBA‐treated nonloaded tibiae versus vehicle‐treated nonloaded tibiae, (*I*) L‐BAIBA‐treated loaded tibiae versus vehicle‐treated nonloaded tibiae, and (*J*) L‐BAIBA‐treated loaded tibiae versus vehicle‐treated loaded tibiae. *n* = 3.

To identify potential mechanisms for increased bone formation in response to sub‐optimal loading and L‐BAIBA treatment, we examined the transcriptional changes in osteocyte‐enriched cortical bone after 2 weeks of 8.25 N loading with vehicle treatment (water), L‐BAIBA treatment without loading, or the combination of 8.25 N loading and L‐BAIBA. RNA from cortical bone was harvested 24 hours after the final bout of loading. Two hundred five genes were significantly regulated by sub‐optimal loading compared to nonloaded, vehicle‐treated bone (Fig. 6*A,E,F* and Supplemental File 1 in Data S1). Among the most significantly upregulated genes were *Wnt1* and *Wnt10b*, activators of Wnt/β‐catenin signaling. Also upregulated were the transforming growth factor β (TGFβ) superfamily members and bone morphogenetic proteins *Bmp2*, *Bmp8a*, and *Bmp8b*. Other upregulated genes included xin actin binding repeat containing 1 (*Xirp1*), bradykinin receptor B2 (*Bdkrb2*), G‐protein‐coupled receptor 3 (*Gpr3*), and *Neu2*. The mineralization regulators dentin matrix protein 1 (*Dmp1*) and the phosphate regulating endopeptidase homolog X‐linked (*Phex*) were also upregulated (Supplemental File 1 in Data S1). The majority of genes downregulated by sub‐optimal loading were histones (21/38, 55%), including H1 linker histones and H2, H3, and H4 core histones (Fig. 6 *A*, Table 1 and Supplemental File 1 in Data S1). The ribosomal RNA precursor *Rn45s* was also significantly downregulated after 2 weeks of loading. GO analysis identified many significantly enriched pathways including TGFβ signaling (GO:0071560; GO:0007179; GO:0071559), muscle cell/tissue development (GO:0060537; GO:0042692; GO:0014706; GO:0051146), ossification (GO:0001503), osteoblast differentiation (GO:0001649; GO:0045667), and biomineralization (GO:0110148) (Fig. 6*G*, Supplemental File 2 in Data S1). GO Cellular Component Ontology identified the enrichment of genes associated with the plasma membrane and dendrite membrane (GO:0098590; GO:0032590) and the chromosome and nucleosome (GO:0061638; GO:0034506; GO:0043505), consistent with the downregulation of histone genes (Supplemental File 3 in Data S1).

**Table 1:** Significantly Downregulated Histone Genes in 8.25 N Loaded, L‐BAIBA Nonloaded, or L‐BAIBA and 8.25 N Loaded Tibiae Compared to Vehicle‐Treated Nonloaded Tibiae After 2 Weeks

Loaded	L‐BAIBA Nonloaded	L‐BAIBA and Loaded
Hist1h1a		Hist1h1a
Hist1h1d		Hist1h1d
Hist1h1e	Hist1h1e	Hist1h1e
Hist1h2ak	Hist1h2ak	Hist1h2ak
Hist1h2an		
Hist1h2bb		
Hist1h2bj	Hist1h2bj	
	Hist1h2bk	
		Hist1h2bl
Hist1h2bm		
Hist1h2bn		
Hist1h3c	Hist1h3c	Hist1h3c
Hist1h3f		
		Hist1h3i
Hist1h4a		
Hist1h4b		
Hist1h4c		Hist1h4c
Hist1h4d	Hist1h4d	
Hist1h4f		Hist1h4f
Hist1h4j	Hist1h4j	
Hist1h4k	Hist1h4k	Hist1h4k
Hist2h2bb		Hist2h2bb
		Hist2h3b
Hist2h4	Hist2h4	Hist2h4
Hist4h4		

Comparison of nonloaded, L‐BAIBA‐treated tibiae with nonloaded vehicle‐treated tibiae, identified 203 significantly regulated genes (Fig. 6*B,E,F* and Supplemental File 1 in Data S1). The most highly upregulated genes included LHFPL tetraspan subfamily member 4 (*Lhfpl4*), basonuclin 2 (*Bnc2*), sodium‐coupled neutral amino acid transporter 4 (Slc38a4), and adhesion G‐protein‐coupled receptor V1 (*Adgrv1*). The neuronal genes neurocan (*Ncan*), lin‐7 homolog A (*Lin7a*), gamma‐aminobutyric acid receptor subunit beta‐2 (*Gabrb2*), MAM domain containing glycosylphosphatidylinositol anchor 2 (*Mdga2*), and microtubule‐associated protein 2 (*Map2*) were upregulated by L‐BAIBA treatment (Figure 6B and Supplemental File 1 in Data S1). Also upregulated by L‐BAIBA treatment were peptidase inhibitor 15 (*Pi15*), proteoglycan 4 (*Prg4*), osteocrin (*Ostn*), and osteoprotegerin (*Tnfrsf11b*). Among the downregulated genes in response to 2 weeks of L‐BAIBA treatment were chondromodulin‐1 (*Lect1*), the collagen subunits *Col9a2*, *Col9a3*, and *Col10a1*, chondroadherin (*Chad*), and chondroadherin‐like (*Chadl*). Several histone genes were also downregulated by the 2‐week L‐BAIBA treatment (9/40, 23%), although fewer than observed with mechanical loading. Pathways shown to be highly enriched by GO analysis included those associated with negative regulation of neurogenesis and axon development (e.g., GO:0050768; GO:0051961; GO:0050771; GO:0030517), neuronal projection and axon guidance (GO:0007411; GO:0097485), regulation of synapses (GO:0007416; GO:0050808; GO:0051963; GO:0097060; GO:0045211), and bone mineralization (GO:0030282; GO0110148; GO:01110149) (Fig. 6*H* and Supplemental File 2 in Data S1). GO Cellular Component Ontology showed that the differentially expressed genes were enriched for the extracellular matrix (GO:0031012), cell periphery (GO:0071944), intracellular organelles (GO:0043231; GO:0043227; GO:000043229), and synaptic and postsynaptic membranes (GO:0097060; GO:0045211) (Supplemental File 3 in Data S1).

### The transcriptional response to 2 weeks of L‐BAIBA combined with sub‐optimal mechanical loading is lower compared to loading alone or to BAIBA alone

The comparison of loaded and L‐BAIBA‐treated tibiae with nonloaded vehicle‐treated tibiae resulted in relatively few differentially expressed genes^(^ ^48^ ^)^ (Fig. 6*C,E,F* and Supplemental File 1 in Data S1). *Wnt1* was among the most upregulated genes, as was *Prg4*. Other upregulated genes included myelin protein zero (*Mpz*), potassium voltage‐gated channel subfamily A member 1 (*Kcna1*), *Bnc2*, transient receptor potential canonical 1 (*Trpc1*), and *Lin7a*. The majority of the downregulated genes were linker and core histones (13/17, 76%). *Col9a2* and *Col2a1* were also significantly downregulated. GO analysis identified pathways enriched for muscle contraction (GO:0043034; GO:0030017; GO:0030016; GO:0043292; GO:0097517), the basal plasma membrane (GO:0016323; GO:0009925; GO:0045178; GO:0016324), cell junctions (GO:0030055; GO:0044291; GO:0005912), and collagen (GO:0005581; GO:0062023; GO:0005583) (Fig. 6*I* and Supplemental File 2 in Data S1). GO Cellular Component Ontology identified the enrichment of genes associated with the nucleosome and chromosome (GO:0000786; GO:0061638; GO:0034506) and the basolateral membrane (GO:0016323; GO:0045178) (Supplemental File 3 in Data S1).

The comparison of tibiae subjected to both L‐BAIBA and load with vehicle‐treated and loaded tibiae also resulted in relatively few differentially expressed genes, with the majority of these being downregulated (19/26) (Fig. 6*D* and Supplemental File 1 in Data S1). The most highly upregulated genes were *Cilp* and *Prg4*, both extracellular matrix‐associated genes. Downregulated genes included *Bdkrb2* and transcription factor AP‐2 epsilon (*Tfap2e*), which were significantly regulated by sub‐optimal loading alone. The low number of differentially expressed genes resulted in the identification of only five significantly enriched pathways by GO analysis, which involved collateral sprouting (GO:0048671; GO:0048670; GO:0048668) and insulin‐like growth factor receptor signaling (GO:0043567; GO:0048009) (Fig. 6*J* and Supplemental File 2 in Data S1). No significantly enriched localizations were identified by GO Cellular Component Ontology (Supplemental File 3 in Data S1).

### Regulation of bone transcriptome by either a single bout of sub‐optimal mechanical loading or short‐term L‐BAIBA treatment

**Fig. 7:**
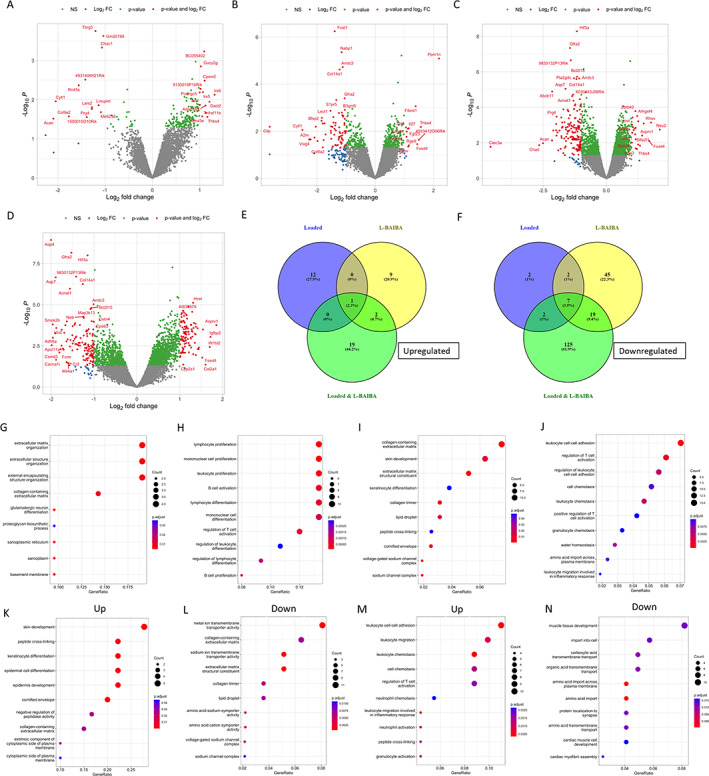
The transcriptional response to a single bout of 8.25 N loading and short‐term L‐BAIBA supplementation. Volcano plots showing significantly regulated genes between (*A*) vehicle‐treated loaded tibiae versus vehicle‐treated nonloaded tibiae, (*B*) L‐BAIBA‐treated nonloaded tibiae versus vehicle‐treated nonloaded tibiae, (*C*) L‐BAIBA‐treated loaded tibiae versus vehicle‐treated nonloaded tibiae, and (*D*) L‐BAIBA‐treated loaded tibiae versus vehicle‐treated loaded tibiae. (*E*) Venn diagram showing number of significantly upregulated and downregulated (*F*) genes between loaded, L‐BAIBA and loaded, and L‐BAIBA nonloaded groups compared to vehicle‐treated nonloaded tibiae. Gene Ontology (GO) analysis of the significantly regulated genes between (*G*) vehicle‐treated loaded tibiae versus vehicle‐treated nonloaded tibiae, (*H*) L‐BAIBA‐treated nonloaded tibiae versus vehicle‐treated nonloaded tibiae, (*I*) L‐BAIBA‐treated loaded tibiae versus vehicle‐treated nonloaded tibiae, and (*J*) L‐BAIBA‐treated loaded tibiae versus vehicle‐treated loaded tibiae. GO analysis of significantly upregulated (*K*) and downregulated (*L*) genes between the L‐BAIBA‐treated loaded tibiae versus vehicle‐treated nonloaded tibiae. (*M*) GO analysis of significantly upregulated and downregulated (*N*) genes between L‐BAIBA‐treated loaded tibiae versus vehicle‐treated loaded tibiae. *n* = 2–3.

Few genes were regulated by loading combined with L‐BAIBA after 2 weeks of treatment, which suggested that the pro‐anabolic gene expression changes induced by the combined treatment may have already occurred by the 2‐week time point. Therefore, we next assessed the transcriptional changes in the tibiae of mice treated with water (vehicle) or L‐BAIBA and subjected to a single bout of sub‐optimal loading to identify the early transcriptional response. In contrast to the gene regulation observed after 2 weeks of sub‐optimal loading, a single bout of sub‐optimal loading in vehicle‐treated mice resulted in few significantly regulated genes^(^ ^26^ ^)^ compared to vehicle‐treated, nonloaded bone (Fig. 7*A,E–F* and Supplemental File 4 in Data S1). Upregulated genes in response to loading included the transcription factors Iroquois homeobox 5 and 6 (*Irx5/6*), *Tnfrsf11b*, disheveled binding antagonist of beta catenin 2 (*Dact2*), and thrombospondin 4 (*Thbs4*). Downregulated genes included the cartilage‐associated extracellular matrix genes aggrecan (*Acan*), cytokine‐like 1 (*Cytl1*), *Col9a2*, and *Prg4*. GO analysis of the differentially expressed genes determined that pathways involved in extracellular matrix organization (GO:1905962) and extracellular structure organization (GO:0043062) were significantly enriched, as was glutamatergic neuron differentiation (GO:1905962) (Fig. 7*G* and Supplemental File 5 in Data S1). GO Cellular Component Ontology showed that the significantly regulated genes were associated with the extracellular matrix (GO:0031012) and external encapsulating structure (GO:0030312) (Supplemental File 6 in Data S1).

Comparison of nonloaded tibiae from vehicle‐treated mice with nonloaded L‐BAIBA‐treated tibiae identified 85 differentially expressed genes (Fig. 7*B,E–F* and Supplemental File 4 in Data S1). *Thbs4* was upregulated, similar to the loaded bone. Other upregulated genes included protein phosphatase Mg^2+^/Mn^2+^ dependent 1 N (*Ppm1n*), the transcription factor Forkhead box d4 (*Foxd4*), and fibroblast growth factor 23 (*Fgf23*). Downregulated genes included cartilage intermediate layer protein (*Cilp*), V‐set and immunoglobulin domain‐containing 4 (*Vsig4*), and alpha‐2‐macroglobulin (*A2m*). *Cytl1* and *Col9a2* were also downregulated by L‐BAIBA, as was observed in the loaded bone. The most significantly regulated pathways by GO analysis involved immune cell regulation (e.g., GO:0046651; GO:0032953; GO:0070661; GO:0042113), in addition to adenylate cyclase‐activated G protein‐coupled receptor signaling (GO:0007189; GO:0007188; GO:0106072) (Fig. 7*H* and Supplemental File 5 in Data S1). GO Cellular Component Ontology showed highly significant enrichment of genes associated with the collagen‐containing extracellular matrix (GO:0062023), cell periphery (GO:0071944), and external encapsulating structure (GO:0030312) (Supplemental File 6 in Data S1).

### The combination of L‐BAIBA with 24‐hour sub‐optimal loading induces a greater transcriptional response than L‐BAIBA or loading alone

Compared with either sub‐optimal loading or L‐BAIBA alone, the combination of loading and L‐BAIBA showed a greater increase in significantly regulated genes versus nonloaded, vehicle‐treated tibiae (175), with the majority of these genes being downregulated (Fig. 7*C,E,F* and Supplemental File 4 in Data S1). As observed for loading alone and for L‐BAIBA alone, the combination resulted in the upregulation of *Thbs4*. *Foxd4* was also increased, similar to L‐BAIBA treatment alone. Genes uniquely upregulated by the combination of loading and L‐BAIBA included neuraminidase 2 (*Neu2*), aspartic peptidase retroviral like 1 (*Asprv1*), ras homolog family member V (*Rhov*), and rho guanine nucleotide exchange factor 4 (*Arhgef4*). *Ctyl1*, *Col9a2*, and *Prg4* showed downregulation by loading and L‐BAIBA similar to that with either treatment alone. Uniquely downregulated genes included C‐type lectin domain family three member A (*Clec3a*), chondroadherin (*Chad*), GDNF family receptor alpha 2 (*Gfra2*), and collagen type 14, alpha 1 (*Col14a1*). Several genes associated with lipid metabolism, perilipin 1 and 4 (*Plin1* and *Plin4*), cell death‐inducing DFFA‐like effector C (*Cidec*), and stearoyl‐CoA desaturase 1 (*Scd1*), were also significantly downregulated by the combination of loading and L‐BAIBA (Supplemental File 4 in Data S1). There was also downregulation of the voltage‐gated sodium channel subunits beta 4 (*Scnb4*) and alpha 7 (*Scn7a*). GO analysis identified pathways involved in collagen‐containing extracellular matrix (GO:0062023; GO0005201), the formation of lipid droplets (GO:0005811), ion transmembrane transporters (GO:0015081; GO:0046873; GO:0005216), amino acid transport (GO:0005283; GO:0005416; GO:0015179; GO:0015171; GO:0005313; GO:0015172), sodium channels (GO:0034706; GO:0005248; GO:0005272), and cell adhesion (GO:0098632; GO:0050839; GO:0098631) (Fig. 7*I* and Supplemental File 5 in Data S1). GO Cellular Component Ontology determined that the significantly regulated genes were enriched for the cell periphery (GO:0071944), extracellular matrix (GO:0031012), and plasma membrane (GO:0005886) (Supplemental File 6 in Data S1).

To identify genes that may contribute to the synergistic effect of L‐BAIBA on sub‐optimal loading, we next compared the differentially expressed genes between the loaded tibiae from L‐BAIBA‐treated mice and the loaded tibiae from vehicle‐treated mice. Two hundred thirty‐eight genes were significantly regulated (Fig. 7*D* and Supplemental File 4 in Data S1). Among the most highly upregulated genes were *Asprv1*, insulin‐like growth factor binding protein 2 (*Igfbp2*), stimulator of chondrogenesis 1 (*Scrg1*), collagen type 2, alpha 1 (*Col2a1*), and *Foxd4*. Downregulated genes included aquaporin 4 and 7 (*Aqp4* and *Aqp7*), *Gfra2*, apolipoprotein L 10a, 11a, and 11b (*Apol10a*, *Apol11a*, and *Apol11b*), and calcium channel, voltage‐dependent, t type, alpha 1i subunit (*Cacna1i*). Many of the significantly enriched pathways identified by GO analysis involved the regulation of immune cells (e.g., GO:0007159; GO:0050863; GO:0050870), which were also enriched by L‐BAIBA treatment alone (Fig. 7*H,J* and Supplemental File 5 in Data S1). Immune cell‐enriched pathways involving chemotaxis and migration (e.g., GO:0030595; 0002523; 0060326; GO:0071621) were also enriched by the combination of loading and L‐BAIBA versus loading alone; however, these pathways were not enriched by L‐BAIBA treatment alone. Other pathways enriched by loading and L‐BAIBA versus loading alone involved water homeostasis (GO:0030104; GO:0050891), amino acid transport (e.g., GO:0089718; GO:0043090; GO:0003333; GO:0006865), regulation of angiogenesis (GO:0045766; GO:1904018; GO:0045765), muscle cell development (GO:0055013; GO:0055006; GO:0055003; GO:0055007), and regulators of cell projections (GO:0031345; GO:0010977) (Supplemental File 5 in Data S1). GO Cellular Component Ontology showed that the significantly regulated genes were highly enriched for the plasma membrane (GO:0005886; GO:0005887; GO0031226; GO0016020) (Supplemental File 6 in Data S1).

To further differentiate the pathways that were significantly regulated by loading and L‐BAIBA, we separated the upregulated and downregulated genes and performed GO analysis. The genes upregulated by the combination of loading and L‐BAIBA versus nonloaded, vehicle‐treated bone were associated with skin development (e.g., GO:0043588; GO:0009913; GO:0008544) and collagen matrix (GO:0062023) (Fig. 7*K* and Supplemental File 7 in Data S1), whereas the downregulated genes were strongly enriched for sodium channels (e.g., GO:0001518; GO:0015081; GO:0046873), collagen matrix (GO:0005581; GO:0062023), and lipid droplets (GO:0005811) (Fig. 7*L* and Supplemental File 7 in Data S1). When comparing between loading and L‐BAIBA versus loading and vehicle, the pathways enriched in the upregulated genes were primarily associated with leukocyte migration and chemotaxis (e.g., GO:0007159; GO:0050900; GO:0030595) (Fig. 7*M* and Supplemental File 7 in Data S1). The downregulated genes were associated with muscle development (GO:0060537; GO:0055003; GO:0014706) and amino acid transport (GO:0089718; GO:1903825; GO:1902475) (Fig. 7*N* and Supplemental File 7 in Data S1).

## Discussion

While exercise is known to be important for maintaining musculoskeletal health, the mechanisms whereby exercise exerts these positive effects are not yet fully defined. Recently, myokines—factors secreted by exercised muscle which determine the beneficial effects of exercise on nonmuscle tissue such as brain, liver, and adipose tissue—have also been identified to promote skeletal health.^(^ ^1^, ^37^, ^38^ ^)^ We previously discovered that L‐BAIBA, a metabolite secreted by contracted muscle, protects against unloading‐induced bone and muscle loss.^(^ ^13^ ^)^ In this study, we investigated the role of L‐BAIBA in the response of bone to enhanced mechanical loading and whether L‐BAIBA could potentiate the anabolic effects of sub‐optimal mechanical loading.

Osteocytes are thought to be the main mechanosensory cells within the bone and respond to mechanical signals through the initial release of small molecules such as PGE_2_, ATP, and NO and the later regulation of genes that control the functions of osteoblasts and osteoclasts.^(^ ^39^, ^40^ ^)^ Loading downregulates genes such as *Sost* and Dickkopf‐related protein 1 (*Dkk1*)^(^ ^24^, ^41^ ^)^ and upregulates the expression of Wnt ligands,^(^ ^41^, ^42^ ^)^ resulting in activation of Wnt/β‐catenin signaling. However, while it is known that osteocytes respond directly to mechanical loading, the contribution of myokines in regulating bone formation during loading is less clear. Irisin, a cleaved peptide released from fibronectin type III domain‐containing 5 (FNDC5) in skeletal muscle, has been shown to have beneficial or negative effects on bone mass, partially through regulating mechanosensitive genes such as *Sost*.^(^ ^11^, ^43^ ^)^ The benefits of L‐BAIBA on maintaining bone mass under hindlimb unloading are independent of *Sost*/sclerostin,^(^ ^13^ ^)^ implicating multiple mechanisms by which myokines can influence bone health.

In this study, L‐BAIBA was only detected in the serum of mice given L‐BAIBA in the drinking water and was not detectable in mice given only water. Interestingly, while the treated mice were given the same concentration of L‐BAIBA (100 mg/kg) in their drinking water, the serum L‐BAIBA levels showed a high degree of variability. This could not be accounted for by L‐BAIBA intake, as the water intake was relatively consistent. The metabolism of L‐BAIBA is not fully understood, but it is known to be converted to L‐valine L‐methyl‐malonyl semialdehyde (L‐MMS) by the mitochondrial enzyme 4‐aminobutyrate aminotransferase (ABAT).^(^ ^44^ ^)^ L‐MMS can be further converted to propionyl‐CoA, a component of the citric acid cycle.^(^ ^45^, ^46^ ^)^ There is also a degree of interconversion between the L and D isoforms of BAIBA^(^ ^47^ ^)^; however, the serum levels of D‐BAIBA were not significantly different between the L‐BAIBA‐treated and control mice. Further understanding of how L‐BAIBA is metabolized is essential in order to be able to regulate serum L‐BAIBA levels.

Although we previously showed that L‐BAIBA protects against disuse‐induced muscle loss,^(^ ^13^ ^)^ the effects of L‐BAIBA on healthy muscle was unknown. The increased grip strength in mice treated with L‐BAIBA suggests an autocrine effect of L‐BAIBA on skeletal muscle that can promote muscle strength and function. While L‐BAIBA in osteocytes is known to function through the MRGPRD receptor,^(^ ^13^ ^)^ the mechanisms behind its effects on muscle are not yet known. Interestingly, another exercise‐induced myokine, neurturin, can improve muscle strength and endurance by regulating motor neuron and neuromuscular junction formation and activity.^(^ ^48^, ^49^ ^)^ Future studies will investigate whether the beneficial effects of L‐BAIBA on muscle are mediated through direct signaling in myocytes or via the neuromuscular junction.

Axial tibial loading of mice is a well‐characterized model to mimic the bone formation response to mechanical strain.^(^ ^50^, ^51^ ^)^ To examine whether L‐BAIBA could synergize with mechanical loading to promote bone formation, we applied sub‐optimal loading forces of 7 and 8.25 N. These values are lower than the force required to initiate bone formation in young, skeletally mature male C57Bl/6 mice (9.4 N^(^ ^26^ ^)^), which allowed us to explore the “threshold‐lowering” effects of L‐BAIBA treatment. Two weeks of L‐BAIBA treatment alone did not induce bone formation, suggesting that, unlike in muscle, L‐BAIBA does not affect basal bone homeostasis. The mice receiving either 7 or 8.25 N of loading also did not have significantly increased bone formation compared to nonloaded tibiae, confirming that these load profiles were sub‐threshold stimuli. We observed a high degree of variability in the 8.25 N loaded tibiae, suggesting that this load profile was likely right at the minimum effective strain (MES) threshold (some mice responded, some did not). There was a significant increase in periosteal bone formation in the mice receiving L‐BAIBA supplementation and 8.25 N tibial loading compared to mice receiving 8.25 N loading alone. This strongly suggests an additive effect of L‐BAIBA on sub‐optimal loading. However, the lack of periosteal bone formation in the L‐BAIBA‐treated mice receiving 7 N of loading indicates that a certain threshold of mechanical strain still must be met in order for L‐BAIBA to be effective.

L‐BAIBA is known to regulate important cellular pathways such as fatty acid and glucose metabolism,^(^ ^12^, ^18^ ^)^ inflammation,^(^ ^52^ ^)^ and the response to oxidative stress.^(^ ^13^ ^)^ However, few genes were significantly regulated after 2 weeks of L‐BAIBA and sub‐optimal loading, which was surprising given the increased bone formation response compared with either loading or L‐BAIBA alone. This is likely due to the transcriptional effects of L‐BAIBA and loading being lost after 2 weeks of treatment, as we found that the number of genes significantly regulated 24 hours after a single bout of sub‐optimal loading with L‐BAIBA supplementation was much greater than the number of genes regulated by either loading or L‐BAIBA supplementation alone. This therefore suggests that an early transcriptional response may underlie the increased bone formation due to loading combined with L‐BAIBA. The loading combined with L‐BAIBA treatment showed high enrichment of extracellular matrix pathways, particularly collagen matrix, as well as genes associated with lipid droplet formation and sodium channels. Further analysis showed that these pathway genes were downregulated compared to nonloaded, non‐L‐BAIBA‐treated bones. The decreased expression of the lipid droplet genes *Plin1*, *Plin4*, and *Cidec* suggests reduced lipid storage. This is consistent with the promotion of fatty acid oxidation, a known function of L‐BAIBA in liver,^(^ ^12^ ^)^ and suggests that changes in bone energy metabolism induced by loading and L‐BAIBA may drive new bone formation.

There was also downregulation of genes associated with voltage‐gated sodium channels (VGSCs), including *Scn4b* and *Scn7a*, in the loaded mice treated with L‐BAIBA. The role of VGSCs in bone has been relatively unexplored compared to the well‐described functions of calcium channels. However, it has been shown that blocking VGSC activity in endothelial cells enhanced their response to fluid flow shear stress.^(^ ^53^ ^)^ Furthermore, it was recently reported that siRNA targeting of the VGSC subunit gene *Scn1a* resulted in increased bone mass in a murine model of senescence.^(^ ^54^ ^)^ Downregulation of VGSC genes due to loading and L‐BAIBA may therefore be an early response to promote increased bone formation.

Compared to the 2‐week combined loading and L‐BAIBA treatment, a larger number of genes were regulated by 2 weeks of loading or L‐BAIBA treatment alone, and most of these were upregulated. In the loading group, the upregulation of *Wnt1* and *Wnt10b* is consistent with the known upregulation of these genes in response to mechanical loading^(^ ^41^, ^42^ ^)^ and the essential role that Wnt/β‐catenin signaling plays during mechanosensation and mechanotransduction.^(^ ^55^, ^56^, ^57^ ^)^ TGFβ/BMP signaling pathway genes were also upregulated by loading, including *Bmp2*, which is known to be regulated by mechanical stress.^(^ ^58^ ^)^ Among the other pathways most regulated by loading were several pathways associated with muscle development and differentiation. The expression of muscle genes in bone, and osteocytes in particular, was previously observed,^(^ ^59^, ^60^, ^61^ ^)^ as has regulation of muscle genes in rat bone after mechanical loading.^(^ ^60^ ^)^ However, the function of these muscle genes in nonmuscle tissue remains elusive.

GO pathway analysis showed that 2 weeks of L‐BAIBA supplementation resulted in the regulation of pathways involved in neurogenesis. There is a distinct morphological similarity between the osteocyte network within bone and that of the neuronal network of the brain. Recently, it was shown that there is also a shared transcriptional profile between neurons and osteocytes.^(^ ^62^ ^)^ The BAIBA receptor Mas‐related G protein‐coupled receptor type D (MRGPRD) is known to be expressed by sensory neurons,^(^ ^63^ ^)^ and bone is highly innervated,^(^ ^64^ ^)^ and this receptor was shown to be responsible for the effects of L‐BAIBA on bone.^(^ ^13^ ^)^ Therefore, future studies will be required to determine whether L‐BAIBA modulates neuronal genes in osteocytes or neurons within bone. There was a highly significant enrichment of lymphocyte‐associated pathways in response to short‐term L‐BAIBA, although the function of these pathways in bone is not yet clear. Several of the genes identified in these pathways have well‐known roles in bone cells, including the Wnt signaling regulator *Lef1* ^(^ ^65^, ^66^ ^)^ and the anti‐inflammatory cytokine *Il‐27*, which is induced in osteoblast and osteocytes during bone remodeling^(^ ^67^ ^)^ and protects against bone loss.^(^ ^68^ ^)^

One of the most striking observations of this study was the downregulation of histone genes in response to 2 weeks of loading in the vehicle‐treated mice. Downregulation of histones was also seen in the L‐BAIBA‐treated mice, although to a lesser extent. While histone modifications have been extensively studied in bone,^(^ ^69^, ^70^, ^71^ ^)^ regulation of histone gene expression has not been reported. The reason for this downregulation is not yet clear, but the reduced histones may allow for chromatin unraveling and the subsequent binding of transcription factors to promote gene transcription. It was recently shown that mechanical stretching of mesenchymal stem cells resulted in increased chromatin accessibility and that these open chromatin regions were associated with binding sites for genes regulating ossification, Wnt and TGFβ signaling.^(^ ^72^ ^)^ A similar effect may be occurring in the loaded bone to allow for activation of these important bone anabolic pathways.

While our findings suggest an important function for L‐BAIBA in potentiating the effects of mechanical loading on bone formation, there are limitations to the study. The serum L‐BAIBA concentrations and bone formation response to L‐BAIBA were variable between animals and such variability may have limited the identification of significantly regulated genes and pathways in the transcriptomics analysis. The experiments were also only performed on young adult male mice. Future studies identifying how sex and age affect the response of bone to mechanical loading and L‐BAIBA treatment are essential to further our understanding of bone and muscle crosstalk during exercise.

In summary, we have shown that an exercise‐induced myokine, L‐BAIBA, can synergize with sub‐optimal mechanical loading to promote new bone formation. This has important implications for the crosstalk between bone and muscle during exercise. L‐BAIBA may be a novel therapeutic strategy to enhance the effects of exercise on both bone and muscle health and to amplify the effects of sub‐optimal resistance exercise in sedentary or immobilized individuals and with space flight.

## Author Contributions

**Matt Prideaux:** Data curation; formal analysis; investigation; visualization; writing – original draft; writing – review and editing. **Alberto Smargiassi:** Data curation; formal analysis; investigation; validation; visualization; writing – review and editing. **Gang Peng:** Data curation; formal analysis; investigation; methodology; visualization; writing – review and editing. **Marco Brotto:** Investigation; methodology; resources; writing – review and editing. **Alexander G. Robling:** Methodology; resources; writing – review and editing. **Lynda F. Bonewald:** Conceptualization; funding acquisition; project administration; resources; supervision; writing – original draft; writing – review and editing.

## Conflict of Interest

The authors declare that they have no conflicts of interest.

### Peer Review

The peer review history for this article is available at https://www.webofscience.com/api/gateway/wos/peer‐review/10.1002/jbm4.10746.

## Supporting information


**Data S1.**Supporting Information.

## Data Availability

The RNA‐seq data from this study are openly available from Gene Expression Omnibus (GEO; GSE214582) at https://www.ncbi.nlm.nih.gov/geo/query/acc.cgi?acc=GSE214582. All other data that support the findings of this study are available from the corresponding author upon reasonable request.
