# Supplementary material for: L‐BAIBA Synergizes with Sub‐Optimal Mechanical Loading to Promote New Bone Formation
Source: JBMR Plus. 2023 Apr 24;7(6):e10746. doi: 10.1002/jbm4.10746 (PMC10241089; doi:10.1002/jbm4.10746)
Supplement: Supplementary file 1 — Data S1.Supporting Information. [file JBM4-7-e10746-s001.docx]

The Supplemental Files containing the differentially expressed genes and GO pathway analysis have been deposited at Mendeley Data and are available at <http://dx.doi.org/10.17632/7ncthkxw26.1>.
